# A Few Observations on the Use of Large Doses of Quinine in the Treatment of Bilious Remittent Fever

**Published:** 1845-12

**Authors:** W. J. Tuck

**Affiliations:** Memphis, Tenn.


*A few Observations on the use of Large Doses of Quinine in the Treatment of Bilious Remittent Fevers.* By W. J. Tuck, M. D., of Memphis, Tenn.—*Messrs. Editors:*—In compliance with a promise made to you some time since, to give you an account of some of the diseases incident to our city, and the mode of treatment, I hasten, amid numerous interruptions, to redeem my pledge to the best of my ability. In doing so I prefer the epistolary mode, as ideas, I believe, are thus expressed with more freedom and ease, and are generally more intelligible to the reader.

And may I not be here permitted to remark, that the subject of fever is *one* of the most, if not *the most, important and interesting* that can engage the attention of the physician, and that, he who can suggest *an idea or remedy*, which would tend to relieve the violence or fatality of this disease, under its various modifications, would confer a benefit upon his species, which would entitle him to the gratitude of all mankind and all posterity. It is estimated by Dr. McIntosh, that *four-fifths* of the deaths that have occurred in the world, have been occasioned by fever. How infinitely important, then, is this subject to the physician, upon whose skill, to a considerable extent, hangs the lives of thousands of his fellow creatures.

Situated, as the city of Memphis is, in the midst of the great valley of the Mississippi, in latitude 36°, in a newly settled region of country, and settled by people from every part of the world, it could not but be expected, that fever in various forms should prevail here more or less; and as this city has been growing so rapidly for the last few years; its population so rapidly increasing, (and there is every prospect of a continuation of this increase and prosperity, from the location of the naval depot at this point, as well as from the great natural advantages of the situation,) any account of the diseases most prevalent in this city, would probably be interesting to the physicians of the South and South-West. Should time and opportunity permit, it would afford me pleasure to contribute to your interesting journal, from time to time, a brief account of the diseases most prevalent in this city, and the most successful mode of treatment. As the season is now approaching when our city is visited, to some extent, with the bilious remittent form of fever, I have thought that it would be appropriate to allude briefly to this disease, and more especially with the view of pointing out the success which has been attained here in the treatment of this form of fever, by the use of quinine; in illustration of which, a few cases, occurring in my own practice, will be presented.

For a number of years past, I presume, it is well known to all the reading physicians of the South, that quinine has been employed to a very great extent, in the treatment of remittent and congestive fevers; and by many physicians, this medicine is regarded as *indispensable* in the treatment of these diseases in the South. Who was the first to adopt this mode of treatment, as far as I am acquainted, has not been ascertained; but, from the success which has attended it, its author, were he known, should be entitled to great praise. The probability is, that whoever first adopted the treatment, was led to do so upon correct and philosophical principles; for, by a very natural process of reasoning, it might be deduced, that, if the bark and quinine were so successful in arresting the most violent forms of congestive or malignant intermittent fever, as occurred in the practice of Lind, Senac, or Bailly, it might be equally successful in the treatment of the *remittent form*, which is produced by the same causes, and which, in some of its forms, may be regarded as congestive fever; for the word *congestive*, according to the best authors, is merely considered a *condition*, and is associated with intermittent, remittent, and typhus fevers, respectively, and gives them a marked character.

The mode of treatment of remittent fevers by large doses of quinine, was not alluded to, so far as I can remember, during an attendance on two courses of lectures, in Philadelphia, in the winters of 1839, and 1840, and the summer of the latter year; and the first time I ever became aware of it, was through the conversation of a young gentleman from Alabama, who was my room-mate, and who had determined to write a thesis upon the success of this mode of treatment, (as he had been convinced of its correctness from the success attending the practice;) from which I attempted to dissuade him, as his views were so contradictory to what I had been taught, and believed to be the opinions of the professors; and I feared that such ultra notions might occasion his rejection. By my own preceptor, a distinguished physician of Virginia, I had been taught, (and this, so far as I was acquainted, was the opinion of the most eminent professors in the country,) that the smallest quantity of quinine would be almost sudden death, if administered in a case of remittent fever. Whether the young gentleman alluded to presented his thesis, I am not aware, as he was not a candidate for graduation until a year after I left. Removing to the South-West in 1840, I had an opportunity of meeting with some distinguished physicians, of enlarged experience, and who, for a number of years, had employed quinine in large doses, in the treatment of bilious remittent fevers, with the most signal success; but my prejudices were so strong, from early education, against such a course, that I could scarcely be made to believe it, until I had an opportunity of witnessing the success with my own eyes during the following summer. In a conversation with Dr. Thomas Fearn, a very distinguished physician of Huntsville, Ala., several years since, he informed me that he was the first person, so far as he was aware, who introduced the use of large doses of quinine in the treatment of a very fatal form of fever, which prevailed in Huntsville, some fifteen years since. He informed me that the disease was producing the most destructive ravages, and was fatal in almost every case. No remedies seemed to be of any avail, but rather did harm, and when any member of a family was seized by the fever, he was regarded as already dead. The doctor mentioned, that he did not recollect what particular circumstances led him to adopt the use of large doses of quinine: but while anxiously casting in his mind to ascertain some more successful plan of treatment to arrest the progress of such a fatal disease, he fell upon the use of large doses of quinine, and the first case in which the treatment was employed, recovered. It was tried with like success in the second case—it was adopted by other physicians; and from that time, the disease was arrested without any difficulty. In connexion with this statement of my own, permit me to extract the following from Dr. Dunglison's Work on New Remedies: "A case of severe remittent has been detailed by Dr. Thomas Fearn, in which he gave, at one dose, three teaspoonfuls, weighing 32 grains. At the end of an hour, there was a diminution of the frequency of the pulse; the inevitable effect of large doses of quinine when its operation is favorable." Dr.

Fearn remarks, that his usual practice in remittent fever, has been to give three doses of twenty grains each, with the interval of an hour between.

But, however this treatment by large doses of quinine originated, or whoever may have been the author, it must be gratifying to every friend of humanity, that so useful a discovery has been made, and that it is now becoming the established practice among the most intelligent physicians of the South; and that where, formerly, death swept over the land with a resistless tide, destroying thousands in his career, we are now able to arrest his destructive march, and almost insure a speedy return of health where, previously, even a hope to live would have been looked upon as folly.

Having briefly alluded to the history of the introdnction (so far as I have been able to inform myself,) of the use of quinine in large doses, in the treatment of bilious remittent fever; and of the reasons by which I was led to adopt the same opinions; I will proceed to give a brief account of the success I have met with in the treatment of this disease in Memphis and vicinity, during the summers and autumn of 1842, 1843, and 1844, and specify a few cases, of which I made very hasty and imperfect notes. My practice during these seasons, has of course been limited, compared with that of older physicians, but, during these three seasons, I think that I have attended, upon an average, from twenty to thirty cases, each year, of remittent fever, and I can state with certainty, that not one of them has died, where the quinine has been used freely, and that only two deaths of the whole number occurred; one, a well marked case of sporadic yellow fever, in which I was afraid to use the quinine, but now believe that if I had done so in the early period of the attack, the man might have been saved; the other, a negro girl, who was brought here very low, with the disease so much advanced, that medicine could produce no effect. Very few cases have occurred in which convalescence did not commence in the course of three or four days, and in a number of cases, the patient would be attending to his business in a week from the time of the attack. Some of the cases would be denominated congestive fever; but correctly speaking should not be called so; since, as has been stated before, congestion only implies a certain condition of disease, and is alike incident to intermittent, remittent, and continued fevers, as well as many other diseases. The cases which I have noted have been done in a very hasty and cursory manner, and will only indicate that quinine has been relied upon as the prominent remedy; although in every case, other means were employed, such as blood-letting, cupping, aperients, &c., as circumstances indicated.

Case 1.—P. B., boy, about 15 years of age; sanguine temperament. Was taken with the usual symptoms of fever on the 26th of June, saw him on the morning of the 28th; had been very sick; said he had taken some pills which operated very freely. Had considerable fever when I saw him; skin dry and hot, with a pain in the head. Quinine was freely employed for three days in quantities of about 15 grains a-day. Cupping, mild aperients, and warm baths were also employed, but the quinine was also relied upon as the prominent remedy. On the fourth day from the time I saw him, all unfavorable symptoms were relieved. On the seventh day he was able to start home, at Holy Springs, some fifty miles distant. There was a high exascerbation of the fever, in this case, in the afternoon, which was entirely checked on the third evening after using the quinine.

CASE 2.—Mrs. J., between 30 and 40 years of age; had been very sick for a week when I was called to see her. Saw her in the morning; pulse very feeble; skin cold, relaxed, and clammy; her extremities cold; I thought her condition very unfavorable; was told she had high fever every afternoon, and was sometimes delirious. Left her quinine to take very freely; did not expect to find her alive next day; directed, also, the use of stimulants. Next morning found her much improved; continued the quinine, with mild aperients, and in a few days she was able to leave her bed, and in a fair way for recovery.

CASE 3.—P. T., a man about twenty years of age, very fleshy. Attacked with a severe chill on July 5th, seized with another on the 7th, when I was called in; high reaction occurred; had taken a large dose of ipecac, which he had procured from the apothecary, and which occasioned great nausea and severe vomiting of large quantities of bile; pulse frequent and full; skin very hot; used bloodletting; and after checking the vomiting by appropriate treatment, gave, in the evening, a mild aperient, and a few grains of Dover's powder. By night, the fever was much abated, and the skin moist. The following morning commenced the use of quinine; gave twelve grains—next morning there was no return of fever. The patient continued to convalesce; and by the continued use of the quinine, *proper regimen*, a dose or so of blue mass and Seidleitz powders, he recovered.

I shall only report one other case, which occurred last summer. On Saturday evening, 24th of August, I was called to see Mr. H——, living fifteen miles in the country. Patient, of a delicate constitution; had suffered much from indisposition during the previous portion of the summer, from the imprudent use of medicines. For several days previously, represented as having a paroxysm every morning; fever continuing through the day; remission at night. When I saw him on Saturday evening, near night, his skin was cool, particularly the extremities; and occasionally cold and clammy perspiration appeared on the forehead; he frequently complained of much pain the stomach; the tongue did not indicate inflammation, yet there was great pain on slight pressure immediately over the *scrobiculus cordis*. With the view of preventing the paroxysm from recurring on the following morning, as I believed the recurrence would probably prove fatal, I gave him during the night at least thirty or forty grains of quinine, until his ears were affected; and applied stimulating applications to the extremities; using also stimulants internally, when the condition of the pulse required them. The patient passed a restless night, talking and muttering most of the time, but towards morning there was a manifest improvement. I neglected to mention, that cups were applied to the epigastrium ; and that opiates were also administered, which seemed to have a good effect, and the patient slept some about daylight. About the time at which his paroxysm usually occurred in the morning, he appeared much better ; reaction had taken place ; skin pleasantly warm; his stomach much relieved and pulse about 75. Being compelled to return to Tennessee, I left directions for the quinine to be continued, with mild cathartics. I heard from the patient on the following Wednesday ; he was much improved ; no return of fever, and no complaint except debility. Prescribed the bark as a tonic.

A number of other cases, where the effects of quinine were equally conspicuous, might be alluded to; but perhaps the above may be sufficient, as I seek brevity; and besides, even such imperfect notes as are given above, were not taken.

The conclusions, then, to which I have been inevitably conducted from my own observations and from the testimony of gentlemen of correct powers of observation, and much more enlarged experience than my own, is, that quinine in free doses, should be relied upon as the prominent remedy, not only in intermittent fever, but in the bilious remittent form, and in what are called the congestive fevers of the South. Other remedies, I believe, in most cases are important, according to the constitution of the patient, the violence of the disease, and other indications which may be presented, which remedies will present themselves to the mind of every intelligent and observing physician ; but no medicine can be compared to the quinine in free doses, for breaking up that train of morbid actions going on in the nervous system, and which, in its own time, brings round a recurrence of a paroxysm so often fatal.

In regard to the theory of the modus operandi of quinine in the treatment of fevers, different opinions have been advanced. My opinion, from the first time I was convinced of the efficacy it possesses, has been, that it acts primarily and directly upon the nervous system, and indirectly, through the medium of this action, upon the sanguineous system. Aware, that there must always be a proper and healthy balance between the nervous and sanguineous system ; the inference was very natural, that quinine produced its salutary effects in arresting the progress of fever, by restoring the impaired energy of the nerves, and thus controlled the circulatory system, and brought about the harmonious balance between these two systems which is so essential to health. In accordance with this theory, and in proof of its correctness, I have, in various instances, witnessed the effects of large doses of quinine in subduing the frequency of the pulse, and relieving severe pains in the head.

In the case of a young gentleman of this town, last summer, the frequency of the pulse diminished from about 140 to 106, in the course of a night, by administering quinine freely. And another remarkable case occurred also last summer, in the person of a lawyer of this place, who had a severe attack of fever. He had a paroxysm and high fever, every afternoon, for some ten or twelve days; the physican in attendance not thinking it appropriate to use quinine in his case. Other physicians were called in, and in our consultation, it was determined that quinine should be exhibited every hour, until thirty grains were taken. There was no return of the paroxysm that evening, or comparatively very slight; and speedy convalescence ensued by a continuance of the quinine in small doses. Instead, then, of fearing to give quinine when there is febrile excitement, hot skin, frequent pulse, headache, &c., as we are taught by the writers on Materia Medica; and looking upon it as a stimulant and irritant, we are forced to the conclusion that this medicine, in large doses, acts as a sedative. And in corroboration of this conclusion, we find the following statement in Stokes' and Bell's practice, which work, however, I had never read until my own observations and experience had led me to the same conviction. The author says, "A large dose acts at once, or very soon, on the nervous system; and by diffusing the sedative influence throughout all its parts, it completely allays irritation, and induces general tranquillity of the functions." And here, in this connection, permit me to make another extract from the excellent work just alluded to, to prove more satisfactorily the sedative effects of large doses of this medicine, and to show that irritation, which is so much dreaded by many, is not produced, even by the most enormous doses which have sometimes been taken by mistake. This work states an instance of a patient who took by mistake a box of pills containing sixty grains of quinine, and with no other inconvenience than a singing in the ears.

I will not protract this letter by any remarks upon this interesting subject. My object has been to give you a plain and brief account of my own experience and observations in the treatment of fever by large doses of quinine; and, by the statement of facts, together with the corroboration of the evidence of others more experienced than myself, to satisfy those members of the profession who are sceptical on this point; that, quinine is by far the best febrifuge we have in the Materia Medica; also, that there is no danger in employing it in what are styled large doses, and that consequently it may be relied upon with more certainty than any other medicine, in the treatment of fever. We are satisfied, that there are many physicians in the South-west who still hold on to the old notions about quinine; who give it only in grain doses, and then only in intermittent fever; and who would not for the world give a grain of it in remittent fever, especially when *the fever is on;* and it is such notions as these that we wish to combat, and to prove by facts and irrefragable evidence, that these notions are entirely incorrect. We believe, that if other gentlemen would write, and every physician who employs large doses of quinine in the treatment of fever, would record and publish the results of his observation and experience, an array of facts and evidence would be presented, which would convince every physician in the country of the truth of our arguments and the correctness of our practice.—*New Orleans Med and Surg. Jour.*

